# Uptake and predictors of direct-acting antiviral treatment for hepatitis C among people receiving opioid agonist therapy in Sweden and Norway: a drug utilization study from 2014 to 2017

**DOI:** 10.1186/s13011-020-00286-2

**Published:** 2020-06-30

**Authors:** Christer F. Aas, Jørn Henrik Vold, Svetlana Skurtveit, Ingvild Odsbu, Fatemeh Chalabianloo, Aaron G. Lim, Kjell Arne Johansson, Lars Thore Fadnes

**Affiliations:** 1 Department of Addiction Medicine, Haukeland University Hospital, Bergen, Norway; 2 Department of Global Public Health and Primary Care, University of Bergen, Bergen, Norway; 3 The Norwegian Institute of Public Health (NIPH), Oslo, Norway; 4 Institute for Clinical Medicine, University of Oslo, Oslo, Norway; 5 Department of Medicine, Karolinska Institutet, Solna, Sweden; 6 Population Health Sciences, Bristol Medical School, University of Bristol, Bristol, UK

**Keywords:** Hepatitis C, Chronic hepatitis C, Treatment uptake, Direct-acting antivirals, Opioid substitution treatment

## Abstract

**Background:**

Treatment with direct-acting antiviral agents (DAAs) offers an opportunity to eliminate hepatitis C virus (HCV) endemic among people who inject drugs (PWID) and people enrolled in opioid agonist therapy (OAT) programs. The objective of this study was to estimate and to compare HCV treatment uptake after the introduction of DAAs among patients receiving OAT in Sweden and Norway. We also aimed to evaluate predictors of DAAs treatment among OAT patients in both countries.

**Methods:**

This observational study was conducted with data from The Swedish Prescribed Drug Register and The Norwegian Prescription Database. We studied dispensed medications to calculate HCV treatment among OAT patients from 2014 to 2017 in Sweden and Norway. HCV prevalence was estimated from primary and secondary sources. Dispensations of medicines from different therapeutic areas, which served as proxy for co-morbidities in 2017, were conditionally adjusted for age, gender, and OAT medications. Logistic regression was used to evaluate these parameters.

**Results:**

In total 3529 individuals were identified with dispensed OAT in the Swedish cohort and 7739 individuals in the Norwegian cohort. HCV treatment was utilized by 407 persons in Sweden and 920 in Norway during the study period. Annual HCV and DAA treatment uptake increased in both countries. The estimated cumulative HCV treatment uptake at the end of 2017 was 31% in Norway and 28% in Sweden. DAA treatment was associated with increased age (aOR 1.8; 95% CI 1.0–3.2) and the dispensation of drugs used for diabetes (aOR 3.2; 95% CI 1.8–5.7) in Sweden. In Norway, lipid modifying agents and antibacterials were associated with decreased odds (aOR 0.4; 95%CI 0.2–0.9, aOR 0.8; 95%CI 0.6–1.0).

**Conclusions:**

An increase in DAA treatment and HCV treatment uptake was observed among Swedish and Norwegian OAT patients whilst introducing new direct-acting antiviral treatment regimens. However, more than two thirds of the OAT population in Norway and Sweden were untreated at the beginning of 2018. A further scale-up is crucial in order to control and eliminate the HCV endemic among OAT patients.

## Background

Treatment of chronic hepatitis C virus (HCV) infection has been subject to vivid changes in the last few years with the introduction of direct-acting antiviral agents (DAAs) [1]. The ambition of any antiviral treatment of HCV infection is elimination of the virus. In that sense, standard treatment prior to 2011 was a combination of pegylated interferon alpha and ribavirin, which saw a sustained virologic response (SVR) in approximately 50 to 56% of patients [1, 2]. SVR is defined as absence of HCV RNA 12 weeks after end of treatment. However, since 2011 various DAAs have become readily available and should make interferon-based therapies almost obsolete. HCV policies including DAA offer countries an opportunity to eliminate HCV endemics, with less side effects, shorter treatment periods and improved adherence as compared to old interferon treatment. Combining two (or three) DAAs have led to a SVR of far beyond 90% also among patients who have been hard to treat in the past [3, 4].

The scale of the HCV endemic among people who inject drugs (PWID) is tragic and is a result of years of failing health policies for vulnerable populations. The HCV prevalence is around 50%, or more, among PWIDs [5, 6], and around 50% among patients on opioid agonist therapy (OAT) [7]. It is estimated that HCV complications will continue to increase within the next few years [8]. DAA treatment has been offered as universal health coverage from 2017 and 2018 in Sweden and Norway, respectively [9, 10]. It seems, however, that the increased accessibility has not benefitted active-PWIDs [11].

The coverage of preventive interventions and harm reduction services varies among PWIDs. Although the distribution of needle and syringe programs is relatively poor [12], opioid treatment programs such as OAT has higher coverage in many countries [13]. OAT has shown to reduce the risk of HCV acquisition [14], and despite ongoing illicit drug use, patients on OAT are achieving high SVR rates [15]. Hence, OAT programs may be a critical intervention for achieving large reductions in HCV transmissions. Several studies have shown that significant reductions in HCV prevalence can be achieved with an adequate increase in HCV treatment uptake [16–18]. Nevertheless, HCV treatment uptake has remained low [19, 20]. In Norway, annual HCV treatment uptake among OAT patients ranged from 1.3 to 2.6% in the period from 2004 to 2013 [20]. HCV treatment uptake, and in particular DAA treatment, among OAT patients in Sweden is unknown. Norway and Sweden share a basic cultural unity, have a comparable socioeconomic and political structure with similar health care systems that are based on the Nordic welfare model [21]. Taking into consideration the potential for HCV disease elimination by publicly funded DAA policies in these countries [9, 13] and the high HCV prevalence among the OAT population, it is essential to calculate the DAA treatment within an OAT delivery platform. Such estimates are important for countries aiming for HCV elimination or endemic control in the near future.

Therefore, this observational study aims to:

1. calculate HCV treatment annually and cumulatively after the introduction of DAAs among patients receiving OAT in Sweden from 2014 to 2017
2. compare DAA treatment between Sweden and Norway among patients receiving OAT from 2014 to 2017 and estimate the HCV treatment uptake
3. evaluate if various dispensed drugs (proxy for comorbidities), age, gender and OAT medication is associated with DAA treatment among OAT patients in Sweden and Norway in 2017

## Methods

### Study design and data sources

This is an observational study among patients on OAT in Sweden and Norway from 2014 to 2017. Data were extracted from The Swedish Prescribed Drug Register and The Norwegian Prescription Database. The registries cover the entire Norwegian and Swedish populations and record all drugs dispensed from pharmacies. All drugs are classified according to The Anatomical Therapeutic Chemical (ATC) classification system [22]. HCV prevalence data is not readily available for Norway and Sweden. Consequently, we employed primary and secondary sources to model HCV prevalence. Data from the INTRO-HCV study in Norway [23] was used in addition to published data on HCV prevalence among a large cohort of Swedish PWIDs [24]. See additional file 1 for a comprehensive description of methodology and data sources.

### Study population and definitions

The study population included all individuals aged 18 to 75 years who received OAT in Sweden and Norway. OAT was defined as being dispensed at least one defined daily dose (DDD) per day per calendar year of buprenorphine, methadone, buprenorphine-naloxone, or levomethadone by summarizing all annually dispensed OAT DDDs divided by 365.25 days.

Moreover, OAT medication per individual was noted as the last dispensation per calendar year. To avoid including other medical indications than OAT, we excluded methadone preparations on the basis of route of administration (injections and tablets), and introduced a dosage criteria in order to make sure that actual patients on OAT were captured. The dosage criteria was set at minimum one DDD daily throughout each calendar year as an inclusion criteria. The study populations were thus chosen annually for both countries and it was possible for an individual to be included in more than one calendar year. See additional file 2 for a flow chart. ATC/DDDs according to 2017 [25] were used to quantify the dispensed OAT medications. A more detailed description of OAT and HCV treatment in Sweden and Norway is provided in additional file 3.

### Calculating HCV and DAA treatment and estimating treatment uptake

HCV treatment was defined as being dispensed either one or more types of pegylated interferon alpha in combination with ribavirin, or one or more of the DAAs per calendar year during the study period (additional file 4). For each country, the annual HCV treatment rates were calculated by dividing the number of individuals with dispensed HCV treatment by the number of individuals on OAT. The cumulative HCV treatment frequency, which is the sum of successive years of treatment, was then calculated as the proportion of patients with dispensed HCV treatment at some point during the study period. Similarly, DAA treatment was calculated by dividing the number of OAT patients with at least one dispensation of DAA by the total number of OAT patients per year, which represents the annual prevalence of DAA use among OAT patients. Using primary and secondary sources, along with several assumptions, as described in detail in additional file 1, we derived a formula to estimate the chronic HCV prevalence in Sweden and Norway as follows;

$$ \boldsymbol{Expected}\ \boldsymbol{N}\boldsymbol{umber}\ \boldsymbol{of}\ \boldsymbol{Chronic}\ \boldsymbol{HCV}=\left(\left(\mathbf{1}-\boldsymbol{\delta} \right)\ast \left[\boldsymbol{\phi} \ast {\boldsymbol{\pi}}_{\boldsymbol{PWID}}+\left(\mathbf{1}-\boldsymbol{\phi} \right)\ast {\boldsymbol{\pi}}_{\boldsymbol{NonPWID}}\right]\ast \boldsymbol{N}\right)-\boldsymbol{\tau} $$

where *N* is the size of the study population, *δ* is the rate of spontaneous HCV clearance, *ϕ* is the proportion of OAT patients who are PWID, *π*_*PWID*_ and *π*_*NonPWID*_ are the anti-HCV prevalence estimates among PWID and non-PWID, respectively, and *τ* is the number of HCV treatments given. Using the above formula, we calculate the expected number of chronic HCV infections in 2014–2017 for Norway and Sweden, with uncertainty in this quantity arising only from the uncertainty in spontaneous clearance. The chronic HCV prevalence was then calculated by dividing the expected number of chronic HCV infections by the total population size in that particular year and setting (i.e. Norway or Sweden). HCV treatment uptake was then estimated by dividing the HCV treatments in each year by the estimated number of chronic HCV infections in that same year, yielding a percentage of chronic HCV infections that were treated per year. The cumulative HCV treatment uptake was then calculated as the sum of HCV treatment uptake across years.

Potential predictors associated with DAA treatment uptake were determined a priori and included OAT medication (methadone/levomethadone vs. buprenorphine-based), age, gender and various dispensed drugs (yes vs. no) from different therapeutic areas that were used as proxies for co-morbidities. All dispensations were recorded at the second ATC level (therapeutic subgroup), except for drugs affecting the nervous system.

### Statistical analyses

All data analyses was conducted in STATA SE 16.0 (StataCorp, TX, USA). Descriptive data was presented as frequencies, percentages, and means, with corresponding 95% confidence intervals where appropriate. Logistic regression was used to estimate whether DAA treatment uptake was associated with gender, age, OAT medication, and dispensations of other drugs in 2017. Statistical significance was set at the *p* < 0.05 level.

### Data handling and ethical considerations

All data were received pseudonymised from registry administrators and subsequently analyzed, therefore, no written consent was obtained from any of the individuals in the study. The study was approved by the Regional Ethical Review Committee in Stockholm, Sweden, (no 2018/2080–31/1) and the Regional Committee for Ethics in Medical Research (no. 2018/939) in Norway. Furthermore, the study was conducted in accordance with the Helsinki Declaration and as an observational study in accordance with international accepted STROBE guidelines [26].

## Results

### Basic characteristics

In Sweden, 3529 individuals receiving OAT were identified. Around 70% were male, with a mean age of approximately 44 years and 45 years in 2014 and 2017, respectively. See additional file 5. The majority of the OAT patients were treated with buprenorphine-based OAT medication (52% in 2014 and 56% in 2017). Altogether 407 individuals in the Swedish cohort received HCV treatment during the study period. In Norway, 7739 individuals were identified during the study period from 2014 to 2017. 70% were male and mean age was 44 in 2014 and almost 46 years in 2017. 55% received treatment with a buprenorphine-based OAT medication in 2017. Altogether 920 individuals in the Norwegian cohort received HCV treatment during the study period (Table 1).

**Table 1:** Basic characteristics of patients receiving OAT in 2014 and 2017 in Sweden and Norway

	*2014*		*2017*	
Country	*Sweden*	*Norway*	*Sweden*	*Norway*
OAT studypopulation, n	2663	6057	2739	5545
Gender, n (%)				
Male	1911 (72)	4266 (70)	1961 (72)	3870 (70)
Female	752 (28)	1791 (30)	778 (28)	1675 (30)
Age, n (%)				
18–35	671 (25)	1219 (20)	647 (24)	878 (16)
36–45	817 (31)	2181 (36)	819 (30)	1747 (32)
46–55	744 (28)	2044 (34)	713 (26)	1998 (36)
56–75	431 (16)	613 (10)	560 (20)	922 (17)
Mean age (SD)				
Male	44 (10)	44.1 (9)	45.1 (11)	46.1 (9)
Female	43.5 (11)	43.1 (9)	44.3 (12)	45.2 (10)
OAT medication, n (%)^a^				
Methadone/levomethadone	1267 (48)	2810 (46)	1198 (44)	2504 (45)
Buprenorphine	875 (33)	2049 (34)	1075 (39)	2190 (40)
Buprenorphine/naloxone	521 (20)	1198 (20)	466 (17)	851 (15)

Sources: The Swedish Prescribed Drug Register (SPDR), The Norwegian Prescription Database (NorPD)

*OAT* Opioid agonist therapy, *SD* Standard deviation

^a^Last registered OAT medication each calendar year

### Estimated HCV prevalence and treatment uptake

**Fig. 1:**
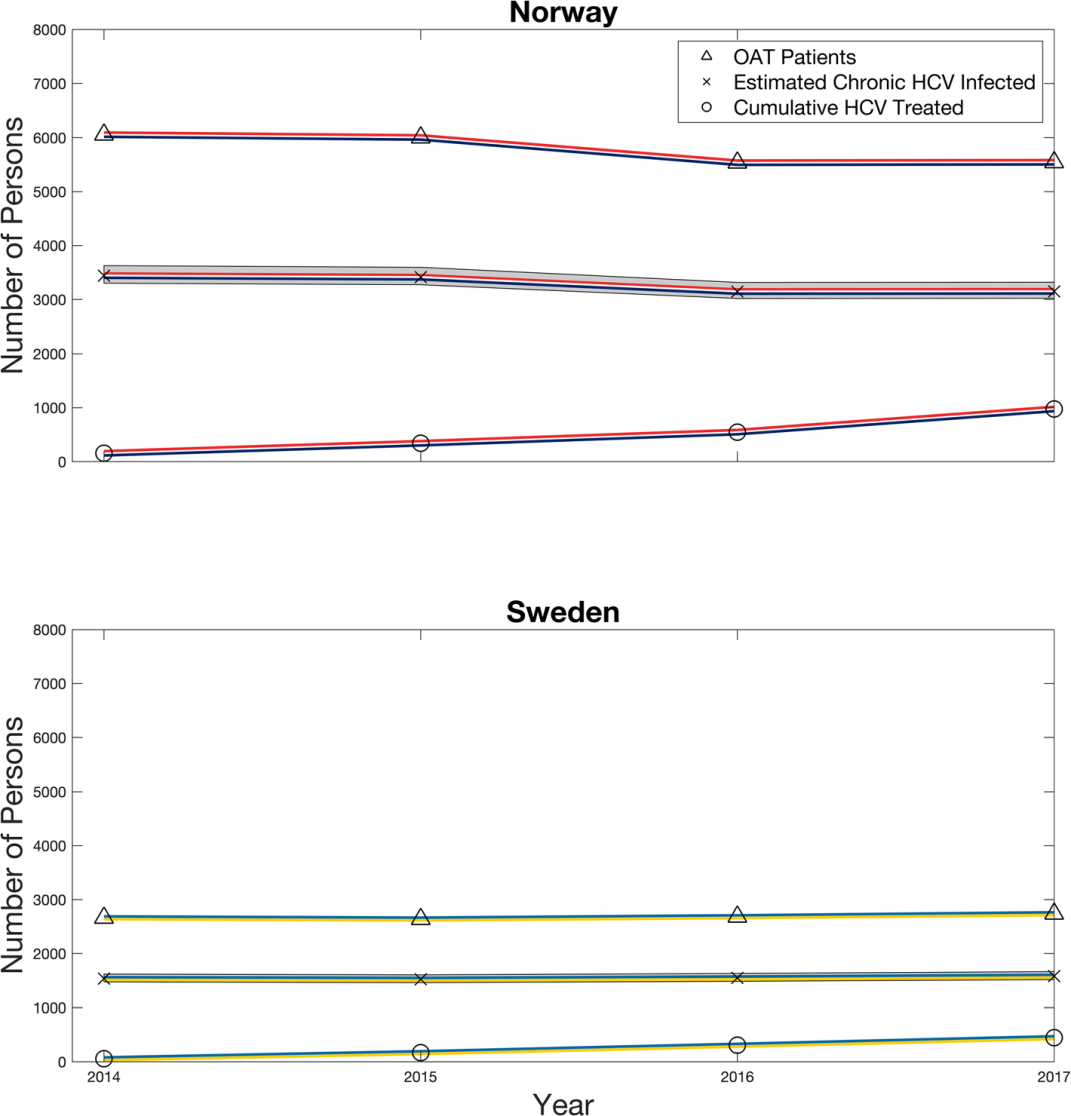
Estimated HCV treatment uptake in Norway and Sweden among OAT patients from 2014 to 2017. HCV = hepatitis C virus infection, OAT = opioid agonist therapy. Sources OAT and HCV treatment: The Swedish Prescribed Drug Register (SPDR), The Norwegian Prescription Database (NorPD). Prevalence: Intro-HCV = Integrated treatment of hepatitis C study, Kåberg et al. [24]: Prevalence of hepatitis C and pre-testing awareness of hepatitis C status in 1500 consecutive PWID participants at the Stockholm needle exchange program, Micallef et al. [27]: Spontaneous viral clearance following acute hepatitis C infection: a systematic review of longitudinal studies. For more comprehensive details on sources and model calculation, see additional file 1.

For Sweden, chronic HCV prevalence was estimated to range from 55.6% (uncertainty interval (UI) 53.3 to 58.8) in 2014, to 53.1 (UI: 50.8–56.3) in 2017. In Norway, prevalence was estimated from 54.4 (UI: 52.1–57.5) in 2014 to 50.0 (UI: 47.7–53.1) in 2017. The cumulative HCV treatment uptake was thus projected to be 31% in Norway and 28% in Sweden for the study period (Table 2). Unadjusted treatment rates for both countries are shown in additional file 6, (Fig. 1).

**Table 2:** Annual and cumulative estimated HCV treatment uptake in Norway and Sweden among OAT patients 2014–2017

	*2014*		*2015*		*2016*		*2017*	
Country	Norway	Sweden	Norway	Sweden	Norway	Sweden	Norway	Sweden
HCV treatment n (overall)	148	54	178	105	216	124	378	124
Study population n,	6057	2663	6005	2640	5537	2683	5545	2739
*HCV treatment % (95% CI)*	*2.4 (2.1–2.8)*	*2.0 (1.5–2.6)*	*3.0 (2.5–3.4)*	*4.0 (3.2–4.7)*	*3.9 (3.4–4.4)*	*4.6 (3.8–5.4)*	*6.8 (6.2–7.5)*	*4.5 (3.8–5.3)*
Expected proportion of OAT patients who are not PWID, n^a^	303	133	300	132	276	134	277	137
Expected Anti-HCV, weighted by PWID status, n^b^	4651	2075	4612	2057	4252	2091	4258	2135
Expected chronic HCV after spontaneous clearance, n (UI)^c^	3442 (3303–3628)	1536 (1474–1619)	3413 (3274–3597)	1523 (1461–1605)	3147 (3019–3317)	1547 (1485–1631)	3151 (3023–3321)	1580 (1516–1665)
Expected chronic HCV after treatment, n (UI)	3294 (3155–3480)	1482 (1420–1565)	3235 (3096–3419)	1418 (1356–1500)	2931 (2803–3101)	1423 (1361–1507)	2773 (2645–2943)	1456 (1392–1541)
Expected chronic HCV after spontaneous clerance and treatment, % (UI)	54.4 (52.1–57.5)	55.6 (53.3–58.8)	53.9 (51.6–56.9)	53.7 (51.4–56.8)	52.9 (50.6–56.0)	53.1 (50.7–56.2)	50.0 (47.7–53.1)	53.1 (50.8–56.3)
***Estimated HCV treatment uptake % (UI)***	***4.5 (4.3–4.7)***	***3.6 (3.5–3.8)***	***5.5 (5.2–5.7)***	***7.4 (7.0–7.7)***	***7.4 (7.0–7.7)***	***8.7 (8.2–9.1)***	***13.6 (12.8–14.3)***	***8.5 (8.0–8.9)***
***Estimated HCV cumulative treatment uptake % (UI)***	***4.5 (4.3–4.7)***	***3.6 (3.5–3.8)***	***10.0 (9.5–10.4)***	***11.1 (10.5–11.5)***	***17.4 (16.4–18.1)***	***19.8 (18.7–20.7)***	***31.0 (29.3–32.4)***	***28.3 (26.7–29.6)***

Sources: The Swedish Prescribed Drug Register (SPDR), The Norwegian Prescription Database (NorPD), Intro-HCV = Integrated treatment of hepatitis C study, Kåberg et al. [24]: Prevalence of hepatitis C and pre-testing awareness of hepatitis C status in 1500 consecutive PWID participants at the Stockholm needle exchange program

Micallef et al. [27]: Spontaneous viral clearance following acute hepatitis C infection: a systematic review of longitudinal studies.

*OAT* Opioid agonist therapy, *HCV* Hepatitis C virus infection, *CI* Confidence interval, *UI* Uncertainty interval

*Anti-HCV* Antibodies to hepatitis C virus, *PWID* People who inject drugs

^a^Expected non-PWIDs among OAT patients set to 5%

^b^Expected Anti-HCV among PWID in Norway 80.8%, expected Anti-HCV among PWID in Sweden 82%, expected Anti-HCV among non-PWID in both Norway and Sweden is 0.7%

^c^Expected spontaneous clearance 26% (22–29%)

For more comprehensive details on sources and model calculation, see Additional file 1

### Dispensations and predictors of DAA treatment in 2017

OAT patients in Norway and Sweden were stratified according to whether they received DAA treatment or not, and compared in 2017. In the Norwegian cohort 366 individuals (6.6%) received DAA treatment whereas in Sweden, 123 (4.5%) individuals received treatment. Variations in treatment within countries were few, except for drugs used for diabetes (Table 3). However, among individuals receiving DAA treatment in Norway, half were also dispensed benzodiazepines compared to only 15% in Sweden. In contrast, 24 and 31% of the Swedish patients treated with DAA also received dispensations of z-hypnotics and antidepressants compared to 15 and 20% in the Norwegian cohort, respectively.

**Table 3:** Dispensed drugs to patients receiving OAT and OAT/DAAs in Norway and Sweden in 2017

Year	*2017*		*2017*	
Country	Norway		Sweden	
OAT study population, n	5543		2739	
	Only OAT	DAA + OAT	Only OAT	DAA + OAT
	5177	366	2616	123
Drugs	No. (%)	No. (%)	No. (%)	No. (%)
Drugs used in Diabetes	197 (4)	14 (4)	161 (6)	18 (15)
Antithrombotic agents	529 (10)	35 (10)	217 (8)	8 (7)
Cardiovascular system drugs ^a^	842 (16)	67 (18)	622 (24)	37 (30)
Lipid modifying agents	271 (5)	10 (3)	121 (5)	4 (3)
Sex hormones and modulators of genital system	654 (13)	51 (14)	430 (16)	14 (11)
Antibacterials for systemic use	1901 (36)	112 (31)	915 (35)	33 (27)
Anti-inflammatory and ant-rheumatic products	1155 (22)	69 (19)	570 (22)	25 (20)
Drugs for obstructive airway diseases	1048 (20)	68 (19)	410 (16)	14 (11)
Benzodiazepines^b^	2368 (46)	181 (50)	402 (15)	19 (15)
Hypnotics and sedatives^c^	797 (15)	54 (15)	691 (26)	30 (24)
Antiepileptics^d^	823 (16)	57 (16)	629 (24)	25 (20)
Antidepressants^e^	960 (19)	73 (20)	1008 (39)	38 (31)
Antipsychotics^f^	1401 (27)	85 (23)	602 (23)	28 (23)

Source: The Swedish Prescribed Drug Register (SPDR), The Norwegian Prescription Database (NorPD). All drugs on ATC Level 2, except under Nervous system. See Supplement Table S2

*OAT* Opioid agonist therapy, *DAA* Direct-acting antiviral agents

^a^C01, C02, C03, C07, C08, C09

^b^N05BA01, N05BA04, N05BA06, N05BA12, N05CD02, N05CD03, N05CD08, N03AE01

^c^N05CF01 and N05CF02

^d^N03AA, N03AB, N03AF, N03AG, N03AX

^e^N06AA, N06AB, N06AF, N06AG, N06AX

^f^N05AA, N05AB, N05AC, N05AD, N05AE, N05AF, N05AG, N05AH, N05AL, N05AN, N05AX

In a logistic regression model (additional file 7), DAA treatment was associated with increased age (adjusted odds ratio (aOR) 1.8; 95% CI 1.0–3.2) and dispensation of drugs used in diabetes (aOR 3.2; 95% CI 1.8–5.7) in Sweden. Dispensations of lipid modifying agents and antibacterials were associated with decreased odds (aOR 0.4; 95% CI 0.2–0.9, aOR 0.8; 95% CI 0.6–1.0) of receiving DAA treatment in Norway. Moreover, being female was associated with decreased odds in both countries (S: aOR 0.6; 95% CI 0.3–0.9, N: aOR 0.8; 95% CI 0.6–1.0).

## Discussion

Amid the hepatitis C endemic among PWIDs and individuals enrolled in OAT programs in Sweden and Norway, the study has revealed a large increase in DAA treatment uptake among OAT patients in both countries from 2014 to 2017. As such, our findings reflect the immense progress, which has been achieved in HCV treatment during the recent years with almost a complete shift from interferon-based treatment to solely treatment with DAAs among OAT patients. The cumulative frequency of HCV treatment in the OAT population between 2014 and 2017 was estimated to be 28 and 31%% in Sweden and Norway, respectively.

Despite substantial increase in HCV treatment uptake in advanced health systems like Sweden and Norway, as found in our study, the treatment uptake is still too low and progress too slow globally [20, 28, 29]. Treatment demand has soared after the introduction of DAAs, especially among former PWIDs [11], while people who are still using drugs actively have seemingly not been fully able to benefit from the increased accessibility [11]. In order to reach universal health coverage of DAAs and elimination of HCV, more efforts are needed in countries. Coverage of DAAs varied substantially across European countries, ranging from 0.6 to 10.2% in 2015 [30]. Restrictions in DAA access policies may explain these variations. Among European countries, England, Hungary, Croatia and Slovakia experienced one of the most restricted access policies to DAA treatment compared to Poland, Ireland, the Netherlands, France and Germany, which had the least restrictions during the study period [31]. Our findings saw Sweden with a greater DAA treatment uptake than Norway in 2015, and roughly in the middle among its European counterparts, similar to the last Scandinavian country, Denmark, at close to 4% [30]. Another reason for the low treatment uptake might be concerns about treatment compliance among PWIDs and OAT patients; however, this seems unwarranted as both good adherence and high SVR rates in this group have been documented in several randomized controlled trials [32, 33].

Arguably, poor treatment uptake of DAAs globally and a hard to reach population opts for countries to consider alternative health service delivery platforms. Addressing barriers to HCV treatment and testing are important. Between 60 and 70% of people enrolled in various opioid treatment programs are offered onsite testing for HCV [29], which is too low. OAT programs could thus benefit from introducing universal HCV testing and linkage to care in OAT settings. Perhaps OAT programs, together with infectious disease and gastroenterology/hepatology specialists, could explore any opportunities for non-specialists to dispense DAA regimens to increase treatment uptake. Psychoeducation to improve knowledge among OAT patients regarding treatment, possible side effects and HCV infection seems to improve both SVR rates and adherence to treatment and should also be considered implemented in an OAT setting [34]. Furthermore, current drug use or any fear of reinfection in patients already treated for HCV should not hinder treatment with DAA. Reinfections seems to be low (1–5%), even if treated patients return to active drug use [35].

The differences between Sweden and Norway are interesting and relevant for other settings. Prevalence of anti-HCV among PWIDs seems consistently higher in Sweden compared to Norway [36, 37]. Coverage of OAT is higher in Norway than Sweden. Waal et al. estimate an overall OAT coverage of around 60% among people with opioid dependence in Norway [38] compared to 10 to 50% OAT coverage in Sweden [39]. Differences in OAT eligibility criteria could explain lower coverage of OAT in Sweden as compared to Norway. Norway altered its OAT guidelines in 2010, making opioid addiction the absolute criteria for inclusion and being retained in treatment, and there is a high threshold for discharging patients from OAT. However in Sweden, current OAT guideline has lower thresholds for OAT cessation in the case of repeated illicit drug use [7, 40]. The two populations may therefore be different and Swedish OAT patients could have less ongoing drug use, which could lower the risk of HCV and increase the chance for HCV treatment success. However, the Norwegian strategy could be more effective at a population level since hard to reach groups are included and illicit drug use is not considered as an exclusion criterion for OAT.

With the provision of DAA treatment available for all Swedish and Norwegian patients, it may be tempting to argue that this is the beginning of the end for the HCV endemic. In addition to OAT, maintaining a high coverage of needle and syringe availability in these countries, together with continued scale-up of DAA treatment, it may be possible to reduce incidence by 90% by 2030 as shown in a modeling study from the UK [41]. On the other hand it may still seem embryonic as there may be shortcomings in current HCV surveillance systems. HCV has been notified to The Norwegian Surveillance System for Communicable Diseases since 1990, yet, there has been no distinction between anti-HCV, HCV RNA or HCV core antigen reporting before 2016 [20]. Hence, accurate HCV prevalence and incidence data prior to 2016 are not readily available. Furthermore, in order to eliminate HCV as a public health threat by 2030, which both countries have embraced, a coherent and structured national plan is essential. The Norwegian Health Ministry introduced a national hepatitis C strategy in 2016, and was later revised in 2018, which focuses on DAA treatment, HCV surveillance, and prevention, and aims to reduce HCV incidence by 90% within 2023 [42]. On the contrary, an ambitious national Swedish hepatitis C plan has not yet been established [43].

Our findings suggest few inter-country differences in dispensed drugs among those treated with DAAs and those not, except for drugs used for diabetes in the Swedish cohort, which was significantly higher and demonstrated a strong association with DAA treatment. Chronic HCV might be a risk factor for developing immune system disorders, heart disease and diabetes, especially diabetes type II as the viral infection may increase insulin resistance [44, 45]. This finding was not mirrored in the Norwegian cohort. Dispensed drugs can serve as a proxy for co-morbidity and it is well-established that both somatic and especially mental illness are underdiagnosed and undertreated among individuals with substance use disorders [46]. This does not explain the vast differences we observed among dispensations of benzodiazepines, z-hypnotics, and antidepressants comparing Sweden and Norway. Older patients are more likely to have cirrhosis and longer HCV treatment courses compared to younger patients. A reason for the observed age difference may be that the younger patients are usually harder to reach due to an unstable life situation and drug abuse related behavior. Similarly, the analyses point toward women being less likely to be treated for HCV, however, this could be due to women being underrepresented in services.

### Strengths and limitations

The national prescription registries capture large populations, and as such, provide researchers with precise and near complete databases. The main strength of this study is that it offers a large sample of OAT patients being treated for HCV.

However, this study has several limitations. As the patients were included each calendar year with a dosage criteria, a patient who commenced treatment late or quit early during the year may not obtain sufficient exposure to be included in that particular year. Moreover, OAT treatment in Norway and Sweden is not uniform. Most individuals are dispensed OAT medications at pharmacies while others receive the drugs at OAT outpatient clinics, which means that those latter patients are not identified in this study. OAT and HCV treatment administered to hospitalized and institutionalized patients are also not recorded in the registries. In addition, DDD does not necessarily reflect the prescribed daily dose.

Furthermore, HCV treatment uptake data was not linked on an individual level to diagnosis codes of HCV according to International Statistical Classification of Diseases and Related Health Problems version 10 (ICD-10) or the International Classification of Primary Care (ICPC), rather, it was estimated from published reports and modelled where adequate data sources were missing. Thus, there is some uncertainty in the denominator of people with HCV in need of treatment. The predictors for DAA treatment were limited to the main dispensed drugs and sociodemographic variables and so did not fully acknowledge that there could be other vital reasons why access to DAAs would be limited in this group of patients.

Finally, PWID are a heterogeneous group of individuals, and one should be careful not to generalize OAT patients to include all PWIDs.

## Conclusion

This study indicates a large scale-up in DAA treatment among Swedish and Norwegian OAT patients. Cumulative HCV treatment uptake was around one-third from 2014 to 2017 in both countries, attributed by a complete shift to DAA treatment regimens. Amidst a HCV endemic among PWIDs, it seems that two-thirds of OAT patients in need of treatment were untreated in the beginning of 2018. Coupled with the prospect of HCV elimination, there is a need for further scale-up of the most effective HCV treatment strategies, by identifying possible predictors of treatment and to establish more accurate surveillance systems in order to provide better care to this group of marginalized people.

## Supplementary information

**Additional file 1. ***Methodology description: estimating chronic hepatitis C (HCV) prevalence among people on opioid agonist therapy (OAT) in Norway and Sweden:*

**Additional file 2. ***Flowchart of study populations in Norway and Sweden from 2014 to 2017*. According to World Health Organization Collaborating Centre for Drug Statistics Methodology, ATC/DDD Index. OAT = opioid agonist therapy, DDD = defined daily dose. The DDDs are the assumed average maintenance dose per day for a drug used for its main indication. *Therapeutic subgroup, chemical subgroup, or chemical substance. **Including clonazepam. ***Excluding clonazepam

**Additional file 3. ***Opioid agonist therapy and hepatitis C treatment in Norway and Sweden*

**Additional file 4. ***Anatomical Therapeutic Chemical Classification and defined daily dose for OAT medications*. OAT = opioid agonist therapy; SD = standard deviation; Sources: The Swedish Prescribed Drug Register (SPDR), The Norwegian Prescription Database (NorPD). *Last registered OAT medication each calendar year

**Additional file 5. ***Basic characteristics of patients receiving OAT from 2014 to 2017 in Sweden and Norway*. OAT = opioid agonist therapy, HCV = hepatitis C virus infection, DAA = direct-acting antiviral agent, CI = confidence interval. Sources: The Swedish Prescribed Drug Register (SPDR), The Norwegian Prescription Database (NorPD). *Exluding Ribavirin (J05AP01)

**Additional file 6. ***Annual and cumulative DAA and HCV treatment among patients receiving OAT from 2014 to 2017*. OAT = opioid agonist therapy, DAA = direct-acting antiviral agent, OR = odds ratio, aOR = adjusted odds ratio, CI = confidence interval. Source: The Norwegian Prescription Database (NorPD) and The Swedish Prescribed Drug Register (SPDR). *N05BA01, N05BA04, N05BA06, N05BA12, N05CD02, N05CD03, N05CD08, N03AE01. **N05CF01 and N05CF02. ***N03AA, N03AB, N03AF, N03AG, N03AX. ****N06AA, N06AB, N06AF, N06AG, N06AX. *****N05AA, N05AB, N05AC, N05AD, N05AE, N05AF, N05AG, N05AH, N05AL, N05AN, N05AX. For ATC codes see additional file 4

**Additional file 7. ***Logistic regression on factors associated with DAA treatment among patients receiving OAT in 2017*. OAT = opioid agonist therapy. Sources: The Swedish Prescribed Drug Register (SPDR) and the Norwegian Prescription Database (NorPD). *Methadone, levomethadone, buprenorphine and buprenorphine-naloxone

## Data Availability

Supplemental tables, figure and data sources in this observational study are available in this published article and its additional files.

## Abbreviations

OAT: Opioid agonist therapy
DAA: Direct-acting antiviral agents
HCV: Hepatitis C virus
PWID: People who inject drugs
NorPD: The Norwegian Prescription Database
SPDR: Swedish Prescribed Drug Register
ATC: Anatomical Therapeutic Chemical classification system
DDD: Defined daily dose
Anti-HCV: Antibodies to the Hepatitis C virus
SVR: Sustained virologic response
INTRO-HCV: Integrated treatment of hepatitis C study
