# Supplementary material for: Uptake and predictors of direct-acting antiviral treatment for hepatitis C among people receiving opioid agonist therapy in Sweden and Norway: a drug utilization study from 2014 to 2017
Source: Subst Abuse Treat Prev Policy. 2020 Jun 30;15:44. doi: 10.1186/s13011-020-00286-2 (PMC7325258; doi:10.1186/s13011-020-00286-2)
Supplement: Supplementary file 1 — Additional file 1. Methodology description: estimating chronic hepatitis C (HCV) prevalence among people on opioid agonist therapy (OAT) in Norway and Sweden: [file 13011_2020_286_MOESM1_ESM.docx]

***Additional file 1***

Methodology description: estimating chronic hepatitis C (HCV) prevalence among people on opioid agonist therapy (OAT) in Norway and Sweden:

Data on prevalence of chronic HCV among OAT patients are not readily available for either Norway or Sweden. We therefore had to make estimation from the best available primary and secondary sources.

We used data from the INTRO-HCV from Bergen and Stavanger as proxy to estimate prevalence among Norwegian OAT patients [1]. This is a nested cohort study linked to the multicenter randomized controlled trial and longitudinal observation study. The data was collected from 2016 to 2019. The main aim of the study is to compare the efficacy of integrated and standard treatment of HCV. The study design is described in detail in the published study protocol [1]. Among 752 patients, preliminary results from blood samples showed a prevalence of anti-HCV of 80.8% and RNA of 44.5%. Anti-HCV, which is antibodies to the hepatitis C virus, indicate current infection, clearance of the infection, or that anti-viral treatment has been successful. HCV RNA indicates current infection. History of injecting drug use was also included. Among OAT patients in the cohort, 4.9% answered they have never injected drugs and we subsequently counted those as non-PWIDs. Based on the assumption that Norway and Sweden hold similar demographics, including drug cultures and behaviors [2], we generalized the proportion of non-PWIDs from the Norwegian cohort to also represent the Swedish cohort. It is assumed that anti-HCV prevalence is 0.7% among non-PWID in both Norway and Sweden, which was derived from estimates of HCV prevalence among adults in the general population from a comprehensive global review of HCV epidemiology from 2014 [3]. In addition, several studies have pointed towards a high prevalence of Anti-HCV among Swedish PWIDs [4]. For the purpose of estimating prevalence among the Swedish patients we used published data from a large cohort of PWIDs in Stockholm, which reported a prevalence of Anti-HCV of 82% [5]. Moreover, a systematic review estimated that spontaneous clearance of HCV occurs in approximately 26% (95% confidence interval 22-29%) of acute HCV infections, with the remaining proportion of cases becoming chronic HCV [6]. The above assumptions enabled us to derive a simple formula to estimate chronic HCV prevalence in Norway and Sweden:

$$\boldsymbol{Expected} Number of Chronic HCV=(\left( 1-\delta\right)*\left[ \phi*\pi_{PWID}+\left( 1-\phi\right)*\pi_{NonPWID} \right]*N)-\tau$$

where $N$ is the size of the study population, $\delta$ is the rate of spontaneous HCV clearance, $\phi$ is the proportion of OAT patients who are PWID, $\pi_{PWID}$ and $\pi_{NonPWID}$ are the anti-HCV prevalence estimates among PWID and non-PWID, respectively, and $\tau$ is the number of HCV treatments given. The cumulative HCV treatment uptake was calculated as the sum of HCV treatment uptake across years.

Our model carries several limitations. We have not adjusted for treatment failure or non-adherence. DAAs have a very high cure rate, however, interferon-based therapies only achieve an SVR of less than 60%. Furthermore, since we used data from high-endemic areas around Stockholm, Bergen and Stavanger we are likely to overestimate HCV prevalence for both countries. The Norwegian Centre for Addiction Research have estimated prevalence of HCV in Norway among OAT patients, based on Anti-HCV and self-reports. [7-10]. Mean prevalence during the study period ranged from 51% in 2014 to 43% in 2017. However, self-awareness of HCV may be low and underreported among PWIDs [5], and thus represent an underestimate of HCV prevalence.

References:

1. Fadnes LT, Aas CF, Vold JH, Ohldieck C, Leiva RA, Chalabianloo F, Skurtveit S, Lygren OJ, Dalgard O, Vickerman P *et al*: **Integrated treatment of hepatitis C virus infection among people who inject drugs: study protocol for a randomised controlled trial (INTRO-HCV)**. *BMC infectious diseases* 2019, **19**(1):943.

2. Selin J, Perälä, R., Stenius, K., Partanen, A., Rosenqvist, P., Alho, H.,: **Opioid substitution treatment in Finland and other Nordic countries: Established treatment, varying practices**. *Sage Journals: Nordic Studies on Alcohol and Drugs* 2015, **32(3)**:311–324.

3. Gower E, Estes C, Blach S, Razavi-Shearer K, Razavi H: **Global epidemiology and genotype distribution of the hepatitis C virus infection**. *Journal of hepatology* 2014, **61**(1 Suppl):S45-57.

4. Han R, Zhou J, Francois C, Toumi M: **Prevalence of hepatitis C infection among the general population and high-risk groups in the EU/EEA: a systematic review update**. *BMC infectious diseases* 2019, **19**(1):655.

5. Kaberg M, Hammarberg A, Lidman C, Weiland O: **Prevalence of hepatitis C and pre-testing awareness of hepatitis C status in 1500 consecutive PWID participants at the Stockholm needle exchange program**. *Infectious diseases (London, England)* 2017, **49**(10):728-736.

6. Micallef JM, Kaldor JM, Dore GJ: **Spontaneous viral clearance following acute hepatitis C infection: a systematic review of longitudinal studies**. *Journal of viral hepatitis* 2006, **13**(1):34-41.

7. Waal H BK, Clausen T, Håseth A, Lillevold PH, and Skeie I.: **SERAF Report: Status 2014, an aging MAR-population?** In*.*: The Norwegian Centre for Addiction Research (SERAF); 2015.

8. Waal H BK, Clausen T, Håseth A, Lillevold PH, and Skeie I.: **SERAF Report: Status report 2015.** . In*.*: The Norwegian Centre for Addiction Research; 2016.

9. Waal H BK, Clausen T, Håseth A, Lillevold PH, and Skeie I.: **SERAF Report: Status 2016.** . In*.*: The Norwegian Centre for Addiction Reserach (SERAF); 2017.

10. Waal H BK, Clausen T, Lillevold PH, and Skeie I.: **SERAF Report: Status 2017. MAR 20 years. Status, evaluations and perspectives**. In*.*: The Norwegian Centre for Addiction Research (SERAF); 2018.
