# Supplementary material for: Uptake and predictors of direct-acting antiviral treatment for hepatitis C among people receiving opioid agonist therapy in Sweden and Norway: a drug utilization study from 2014 to 2017
Source: Subst Abuse Treat Prev Policy. 2020 Jun 30;15:44. doi: 10.1186/s13011-020-00286-2 (PMC7325258; doi:10.1186/s13011-020-00286-2)
Supplement: Supplementary file 3 — Additional file 3. Opioid agonist therapy and hepatitis C treatment in Norway and Sweden [file 13011_2020_286_MOESM3_ESM.docx]

***Additional file 3***

Opioid agonist therapy and hepatitis C treatment in Norway and Sweden:

Norway and Sweden have similar welfare and national healthcare systems where the standard is open access to health care for all inhabitants. As such, and in theory, all residents with a diagnosed opioid dependence according to International Classification of Diseases have free access to opioid agonist therapy (OAT) in Norway, however in Sweden, a diagnosis for at least 12 months is required for treatment entry [1, 2]. Pharmacotherapy with either buprenorphine or methadone, in an integrated program with psychosocial support is the mainstay of treatment even though there are vast intercountry differences to this approach [1, 3, 4].

Whereas the first methadone maintenance treatment program dates back to the 1960s in Sweden, modelled after the Dole and Nyswander program, Norway first implemented OAT in 1998 into the general health and social security system [2, 5]. In both countries, inclusion into the program was strict and thus characterized as high threshold and restrictive. However, this practice was abolished in Norway according to the new OAT guidelines from 2010. Since then, entry requirements have been minimal where opioid dependence has been the absolute admittance criteria, the whole OAT program has expanded and taken over by the specialist health services, and patients were no longer subject to involuntary termination based on e.g. illicit substance use [2, 4, 6, 7]. This is not the case in Sweden. Even if admittance criteria are currently less strict compared to previous ones [5], in cases of repeated illicit substance use while receiving OAT, the provision of OAT may be ceased and patients referred to other types of treatment [8, 9].

There are currently around 7500 individuals on OAT in Norway and 4400 in Sweden [8, 10]. Attempts to estimate OAT coverage among people with opioid dependence have proven difficult due to the criminal nature of illicit drug use, difficulties in identifying injecting drug users and people in need of treatment. However, OAT coverage is most likely higher in Norway with around 60% [11].

In both countries HCV testing has been scarce and unsystematically in the national OAT programs. Only in parts of western Norway, as part of the multicenter Integrated treatment of hepatitis C study, all OAT patients have been offered systematically testing for hepatitis C and examined with elastography as part of an annual OAT health assessment since 2017 [12]. Standard hepatitis C testing and treatment are typically provided from an infectious (or gastroenterology) polyclinic attached to a central hospital setting in both countries [13, 14]. Despite open access to health care, treatment with direct-acting antiviral agents have been limited by stage of liver fibrosis during the study period. Only from February 2018 and October 2017, in Norway and Sweden respectively, interferon-free treatment with direct-acting antiviral agents have been offered to all patients with chronic hepatitis C regardless of stage of liver fibrosis [15, 16]

References:

1. Gedeon C, Sandell M., , Birkemose I., , Kakko, J., Rúnarsdóttir, V., Simojoki, K., Clausen, T., Nyberg, F., Littlewood, R., and Alho, H.,: **Standards for opioid use disorder care: An assessment of Nordic approaches** *Sage Journals: Nordic Studies on Alcohol and Drugs* 2019, **36 issue: 3, page(s): 286-298**

2. Waal H CT, Håseth A, Lillevold P.,: **MAR 10 years in Norway. Status report 2008.** In*.*: The Norwegian Centre of Addiction Reserach (SERAF); 2009.

3. (NBHW) TSNBoHaW: **Läkemedelsassisterad behandling vid opiatberoende. Slutsatser och förslag**. In*.*; 2015.

4. Ministry of Health and Care Services: **National guidline for medicaly assisted rehabilitation (MAR) for opioid dependence**. In*.*: The Norwegian Ministry of Health and Care Services; 2010.

5. Ledberg A: **Mortality related to methadone maintenance treatment in Stockholm, Sweden, during 2006-2013**. *Journal of substance abuse treatment* 2017, **74**:35-41.

6. Waal H: **Merits and problems in high-threshold methadone maintenance treatment. Evaluation of medication-assisted rehabilitation in Norway 1998-2004**. *European addiction research* 2007, **13**(2):66-73.

7. Waal H BK, Clausen T, Lillevold PH, and Skeie I.: **SERAF Report: Status 2017. MAR 20 years. Status, evaluations and perspectives**. In*.*: The Norwegian Centre for Addiction Research (SERAF); 2018.

8. European Monitoring Centre for Drugs and Drug Addiction: **Sweden, Country Drug Report 2019**. 2019.

9. The Swedish National Board of Health and Welfare (Socialstyrelsen) **Uppföljning av föreskrifter och allmänna råd om läkemedels-assisterad behandling vid opioidberoende (LARO)**. In*.*; 2017.

10. Waal H BK, Clausen T, Lillevold PH, and Skeie I.: **SERAF RAPPORT 3/2018: Statusrapport 2017. LAR 20 år. Status, vurderinger og perspektiver**. 2018.

11. Nilsen L.: **Færre nye rusmisbrukere inn i LAR**. In: *Dagens Medisin.* Oslo, Norway; 2017.

12. Fadnes LT, Aas CF, Vold JH, Ohldieck C, Leiva RA, Chalabianloo F, Skurtveit S, Lygren OJ, Dalgard O, Vickerman P *et al*: **Integrated treatment of hepatitis C virus infection among people who inject drugs: study protocol for a randomised controlled trial (INTRO-HCV)**. *BMC infectious diseases* 2019, **19**(1):943.

13. DNLF: **Faglig veileder for utredning og behandling av hepatitt C hos voksne**. 2017.

14. Folkehälsomyndigheten: **Hälsofrämjande och förebyggande arbete med hepatiter i Sverige - Kunskapsunderlag, analys och bedömningar**. 2019.

15. Lagging M, Wejstal R, Duberg AS, Aleman S, Weiland O, Westin J: **Treatment of hepatitis C virus infection for adults and children: updated Swedish consensus guidelines 2017**. *Infectious diseases (London, England)* 2018, **50**(8):569-583.

16. Ministry of Health and Care Services: **Hepatitt C skal utryddes som folkehelseproblem** 2019.
